# Estimating the cost of young stock mortality in livestock systems—An application to sheep farming in Ethiopia

**DOI:** 10.3389/fvets.2024.1389303

**Published:** 2024-07-24

**Authors:** Joachim Otte, Christian Schnier, Fiona K. Allan, Gareth Salmon, Johanna T. Wong, Bruno Minjauw

**Affiliations:** ^1^Food and Agriculture Organization (FAO), Rome, Italy; ^2^Supporting Evidence Based Interventions-Livestock (SEBI-Livestock), Royal (Dick) School of Veterinary Studies, University of Edinburgh, Easter Bush Campus, Midlothian, United Kingdom

**Keywords:** young stock mortality, bio-economic modeling, cost-benefit, sheep, Ethiopia

## Abstract

**Introduction:**

In sub-Saharan Africa, pre-weaning young stock mortality (YSM) is in the order of 20 to 30% across most livestock species and production systems. High YSM has significant economic implications for livestock keepers, but few studies provide estimates of the “cost of YSM.” This study explores a bio-economic herd modeling approach to estimate the “cost of YSM” at farming/livestock system level.

**Methods:**

The static zero-growth version of DYNMOD was used to calculate the annual physical and monetary output of a sheep flock consisting of 100 breeding females at different levels of lamb mortality. Production parameter values and prices were taken from recently published research. Calculations were carried out for values of lamb mortality decreasing from 30% to 0% in 5% intervals, with 20% representing the “baseline” YSM. Calculations were repeated for a “high” fertility scenario (100% vs. 59% parturition rate) to gauge the sensitivity of the cost of YSM to another parameter determining flock productivity.

**Results:**

The relation of revenue per head and YSM is close to linear over the range of analyzed YSM with 1% decrease in YSM resulting in an increase in revenue per animal of approximately 1%. At the higher fertility rate, the absolute cost of YSM to sheep farmers is higher while the relative increase in revenue per animal resulting from YSM reduction is lower. The estimated difference in revenue of the 100-ewe flock between the 20% and 0% lamb mortality scenarios (at baseline fertility) amounts to approximately USD 90 per additionally surviving lamb, which is far above its market value.

**Discussion:**

Reduced lamb mortality ultimately impacts flock revenue through increased sales of “mature” animals, which embody the value of a lamb plus the revenue/profit from raising it to marketable age/weight. The modeling results suggest that foregone profit is an important component of the systemic “cost of YSM.” Consequently, expected profit per animal, in addition to its current market value, is essential for estimating the absolute cost of YSM at farming system level.

## 1 Introduction

Young stock mortality (YSM) refers to the untimely death of young livestock, such as cattle and camel calves, lambs, and kids. In sub-Saharan Africa, where livestock often serve as a primary source of income, food, and livelihood, pre-weaning YSM has been found to be in the order of 20 to 30% across most livestock species, production systems and agro-ecological zones (1–6). High YSM has significant economic and social implications for livestock keepers and numerous studies have been conducted to identify its causes (7–9). Far fewer studies have explored interventions to reduce YSM and even less provide information on the costs and benefits of tested interventions. A prerequisite for any cost-benefit analysis of YSM reduction is an estimate of the “cost” of YSM. Various studies have estimated the cost of YSM by multiplying the number of young stock dying by the “potential value” (10), “probable market value” (11), or “cost of production” of the deceased animal (12). This approach has several limitations: (i) as young stock are rarely traded before weaning, it is difficult to assign a value to these animals, particularly if death occurs shortly after birth, (ii) it does not consider foregone profit had the animal survived, and (iii) “secondary” effects of YSM are not accounted for, such as, possible reduction or even cessation of milk production of the dam and/or changes in herd structure, which may lead to relative shifts in income streams.

The aim of this (desk) study is to explore an alternative approach to estimate the cost of YSM at farming system, as opposed to individual farm-level. Taking a farming systems perspective avoids the need to consider between-farm transactions in response to YSM as these ultimately are a zero-sum game. The approach builds on bio-economic livestock population modeling to assess the impact of changes in animal performance on revenue streams (e.g., meat, milk, hides, and manure) and population composition. We apply the approach to a case study of lamb mortality in Ethiopian sheep flocks drawing on data from published research.

## 2 Materials and methods

The static zero-growth (STEADY2) version of DYNMOD (13); [available at https://gitlab.cirad.fr/selmet/livtools/dynmod/-/blob/master/dynmod_steady2.xlsx?ref_type=heads] was used to calculate the annual physical and monetary output of a sheep flock consisting of 100 breeding females (females >1 year old) and the associated lambs (birth to 6 months old), sub-adults (6 to 12 months old), and male breeders (males >1 year old), at different levels of lamb mortality. STEADY2 adjusts annual offtake rates of male and female adults to maintain flock size constant at the selected level of lamb mortality. This avoids having to value flock inventories at the beginning and end of the year as all output is monetized (14). The approach thus compares the productivity of flocks in equilibrium, i.e., a “steady state,” but does not consider transition costs of moving from one state to another.

Production parameter values and prices are taken from Jemberu et al. (15), who used DYNMOD to estimate sheep and goat production in Ethiopia, distinguishing between pastoral and mixed crop-livestock (MCL) production systems. This case study uses the “most likely” parameter values and prices for sheep in MCL systems (Supplementary Table S1), except for lamb mortality and adult offtake rates. The former was set at 20% instead of 18% to simplify the sensitivity analysis. Adult offtake rates were generated by DYNMOD STEADY2 to maintain herd size constant. This contrasts with Jemberu et al. (15), who used the STEADY1 version of DYNMOD, which allows for constant flock growth. Also in contrast to Jemberu et al. (15), this analysis omits income from sheep skins, as sheep are usually marketed as live animals, but includes estimates of flock dry matter feed requirements. The latter are estimated as 2.6% of live weight based on the study “Estimating Disease Burden of Small Ruminants in Ethiopia” available online at: https://animalhealthmetrics.org/estimating-disease-burden-of-small-ruminants-in-ethiopia-application-of-the-global-burden-of-animal-diseases-gbads-framework/. The value of offtake, referred to as “revenue” in the following, was converted from Ethiopian Birr into USD at an exchange rate of 43 Birr to 1 USD. Calculations were carried out for values of lamb mortality decreasing from 30% to 0% in 5% intervals. Results of the zero-growth approach were compared to an approach that keeps herd growth steady at 10.2% [resulting from application of offtake rates from Jemberu et al. (15) at 20% YSM] with changes in inventory value contributing to flock revenue. This approach relies on manual increase of offtake rates as YSM decreases.

The total annual value of output (animals and manure) was converted to a per animal, per ewe, and per 100 kg feed dry matter figure. The difference in revenue per animal between a selected level of YSM and zero YSM was taken as the “gross” cost of the selected level of mortality. Drawing on Legesse et al. (16), “net” costs of YSM were subsequently estimated assuming that variable costs (e.g., feed, veterinary inputs, hired labor) account for between 15% and 25% of the annual per animal revenue. Finally, all calculations were repeated for a “high” fertility scenario (100% parturition rate) to gauge the sensitivity of the cost of YSM estimates to another parameter determining offtake potential at constant flock size.

## 3 Results

### 3.1 Baseline production and annual revenue

The results of the simulation of flock demographics and output at “baseline” lamb mortality risk (20%) and parturition rate (59%) are presented in Table 1. At the 20% lamb mortality risk, the simulated sheep flock mainly consists of female breeders (59%), can sustain an overall offtake rate of 30% (50.9/169.6) and generates an annual revenue of USD 24.5 per head (USD 41.5 per adult ewe, USD 11.2 per 100 kg feed dry matter). At assumed variable costs of 15% and 25% of revenue, annual gross margin per head would amount to USD 20.8 and USD 18.4. Revenue from the sale of manure contributes < 5 percent of total revenue, and nearly half (46.2%) of the revenue from animal sales stems from the sale of adult/breeding females. Approximately 12 lambs are predicted to die over the course of a year, representing nearly 60% of total annual animal losses.

**Table 1:** Simulated demographics and offtake of a sheep flock of 100 breeding females using most likely production parameter values and prices from Jemberu et al. (15) at 20% (“baseline”), 10% and 0% lamb mortality risk.

	**20% Lamb mortality**		**10% Lamb mortality**		**0% Lamb mortality**	
	**Nr**	**%**	**Nr**	**%**	**Nr**	**%**
**Inventory**						
Adult/breeding females	100.0	59.0	100.0	57.4	100.0	55.9
Adult/breeding males	10.5	6.2	10.5	6.0	10.5	5.9
Other females	31.6	18.6	34.2	19.6	36.8	20.6
Other males	27.5	16.2	29.6	17.0	31.7	17.7
**Total**	169.6		174.3		179.0	
**Deaths**						
Adults/breeders	5.4	25.4	5.4	34.5	5.4	54.4
Sub-adults	3.6	17.0	4.1	26.0	4.5	45.6
Lambs	12.3	57.6	6.2	39.5	0.0	0.0
**Total**	21.3		15.7		9.9	
**Live animal offtake**						
Adult/breeding females	23.5	46.2	27.0	46.5	30.4	46.8
Adult/breeding males	10.0	19.6	11.4	19.7	12.8	19.8
Sub-adult females	1.2	2.4	1.3	2.3	1.5	2.3
Sub-adult males	16.2	31.8	18.2	31.4	20.2	31.1
Lambs	0.0	0.0	0.0	0.0	0.0	0.0
**Total**	50.9		57.9		65.0	
**Offtake value (USD)**						
Live animals	3,973.3	95.8	4,526.8	96.2	5,080.1	96.5
Manure	176.3	4.2	179.7	3.8	183.0	3.5
Total	4,149.7		4,706.4		5,263.1	
Per head	24.5		27.0		29.4	
Per breeding female	41.5		47.1		52.6	
Per 100 kg feed DM	11.2		12.5		13.7	

### 3.2 Effect of YSM on annual revenue

**Figure 1:**
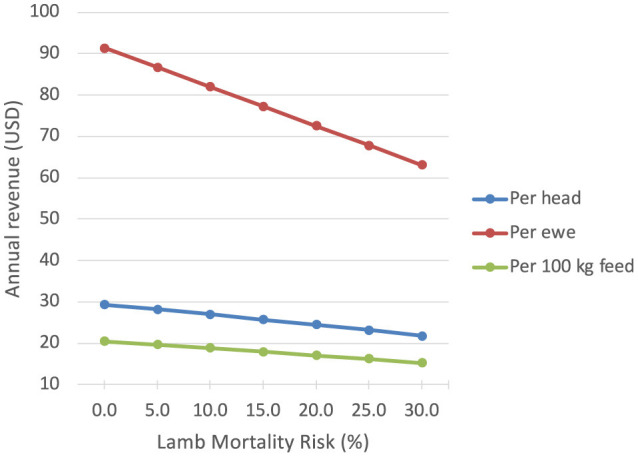
Relationship between lamb mortality risk and annual revenue per head, ewe, and 100 kg feed dry matter.

At zero YSM, the estimated annual revenue per head amounts to USD 29.4, an increase of USD 4.9 or 20% over the 20% YSM baseline scenario, while annual revenue per ewe increases to USD 52.6 (or 26%) and revenue per 100 kg feed rises to USD 13.7 (22%) (Table 1). The relationships between lamb mortality risk and revenue per head, ewe, and 100 kg feed dry matter are displayed in Figure 1. The relationship of revenue per head and YSM is close to linear over the range of analyzed YSM with one percentage decrease in YSM resulting in an increase in revenue per animal of approximately USD 0.24 (or app. 1%). If variable costs are not considered, the USD 4.9 per sheep would represent the cost of YSM to Ethiopian sheep farmers engaged in mixed crop-livestock production. At assumed variable costs of 15% and 25% this figure decreases to USD 4.2 and USD 3.7, respectively. The relationship between annual revenue per 100kg feed, likely a key element of the variable production costs, and lamb mortality closely aligns with the relationship between lamb mortality and annual revenue per head, while the relative increase in revenue per ewe with decreasing lamb mortality is considerably higher.

The constant growth approach yields very similar results to those of the zero-growth approach with predicted annual revenues of USD 24.2 per head, 40.3 per ewe, and 11.0 per 100 kg feed at baseline and revenues of USD 28.4 per head, 50.4 per ewe, and 13.1 per 100 kg feed at 0% YSM (Supplementary Table S2).

Closer examination of per animal revenue gains/losses from changes in YSM across a range of values from 0% to 30% reveals that returns to YSM reduction diminish as initial YSM decreases. For instance, reducing YSM from 20% to 15% increases revenue by USD 1.28% or 5.22%, while a reduction from 5% to 0% increases revenue by USD 1.16% or 4.12% (Supplementary Table S3).

### 3.3 Sensitivity of YSM cost to parturition rate

All preceding calculations were repeated for a sheep flock of 100 female breeders with a parturition rate of 100% while all other parameters were left unchanged. At the same 20% YSM, the flock with the higher parturition rate has a lower share of breeding females (48%), can sustain an overall offtake rate of 43% (90.0/210.6) and generates revenues of USD 34.4 per head, USD 72.5 per ewe, or USD 17.1 per 100 kg feed dry matter (Supplementary Table S4). These revenue estimates are 40%, 75%, and 53% above those of a flock with the 59% (baseline) parturition rate. At this higher fertility rate, reducing YSM from 20% to 0% increases returns per animal by USD 5.86 (Supplementary Table S5), which is USD 0.96, or 20%, more than in the baseline scenario. Thus, the absolute cost of YSM to sheep farmers increases at higher levels of reproduction. On the other hand, the relative increase in revenue per animal resulting from YSM reduction is lower in the higher fertility flock, e.g., YSM reduction from 20% to 0% increases revenue by 17.2% in the higher fertility scenario vs. 20.3% in the baseline case. With regards to changes in revenue per kg feed, the respective figures are 19.7% and 22.4%, i.e., very similar to those of revenue per head.

## 4 Discussion

Our use of DYNMOD, a generic bioeconomic herd model, to estimate the cost of YSM to Ethiopian sheep farmers is similar to the modeling approach employed by Bruce et al. (17) to estimate the impact of lamb and ewe mortality associated with dystocia on Australian and New Zealand sheep farms. Bruce et al. (17) used the MIDAS model (Model of an Integrated Dryland Agricultural System), which is context-specific, very detailed, and includes production costs. It, therefore, allows direct estimation of the impact of mortality on farm profit in addition to its impact on physical output and revenue. In contrast to the analysis of Bruce et al. (17), our assessment of the cost of YSM in Ethiopia should be regarded as exploratory. To this end, we preferred the zero-growth (STEADY2) approach over the constant-growth (STEADY1) approach as the latter relies on manual adjustment of offtake rates to maintain flock growth at the desired level and involves assigning prices to animals that are not ready for sale. Both approaches, however, yield very similar results and support the same conclusions.

In the simulated case, reduced lamb mortality ultimately impacts flock revenue through increased sales of “mature” animals, which embody the value of a lamb plus the revenue/profit from raising it to marketable age/weight. The estimated USD 1,113 difference in revenue of the 100-ewe flock from live animal sales between the 20% and 0% lamb mortality scenarios is far above any “market” value that might be assigned to the 12.3 additional surviving lambs. Taking the value of USD 28.5 per lamb from Jemberu et al. (15), which is high for lambs dying in the first month of life when most mortality occurs, lamb mortality loss would amount to USD 351. The large difference between the two estimates of the “cost” of 20% YSM in the same 100-ewe flock suggests that foregone profit, as a sizeable proportion of revenue, is an important component of the “cost of YSM” at system level, i.e., where the loss of an animal cannot simply be mitigated by purchase of a live replacement from another farmer. This finding is in line with the widely applied approach to disaster damage and loss assessment which considers the “value of assets lost” as “damage” and the “revenue that would have been generated with the damaged assets” as “losses” (18).

Consequently, expected profit per animal, in addition to its current market value, is essential for estimating the absolute cost of YSM. Unfortunately, assessment of production costs in (semi-) extensive, low-input, production systems, where animals obtain much of their feed from communal grazing areas, household waste, and crop residues, and family members provide most, if not all, labor input, is rarely attempted and the valuation of inputs, e.g., family labor, is often controversial. However, even though accurate estimates of profit per animal remain elusive, it appears safe to assume that in low-input livestock production systems production costs only account for a small proportion of the revenue generated per animal and that the gains in revenue in Table 1 reflect proportional gains in enterprise profit. In situations where feed costs are the dominant production cost, returns per kg feed could serve as an alternative measure of flock productivity at different levels of YSM. In the simulated case, a 1% decrease in YSM results in an increase of around 1% for any of these metrics.

The cost of YSM is not only determined by the level of YSM, but also by the reproductive performance of the breeding flock. The same YSM results in higher costs as flock reproductive performance increases because the absolute number of lambs dying is higher. However, the relative impact of reducing YSM decreases with increasing flock fertility as the proportional increase in offtake rate which maintains flock size constant diminishes. Reducing YSM also faces diminishing returns with lower levels of initial YSM. This is because the same additional number of survivors represents a smaller increment at higher initial number of survivors (e.g., moving from 80 to 90 survivors is a 12.5% increase while moving from 90 to 100 survivors only represents an 11.1% increase).

Although the estimates of YSM cost depend on initial conditions and do not include production costs, they provide some guidance as to the level of investment in YSM reduction that would yield positive returns. As 0% YSM is unrealistic and returns on reducing YSM diminish as actual YSM decreases, the “cost of avoidable YSM” is more relevant for investment decision-making. In Ethiopia, the Young Stock Mortality Reduction Consortium (YSMRC) has demonstrated that YSM can be reduced to 5–10% through implementation of “intervention packages” (19, 20). We estimate that reducing YSM in sheep from 20% to 10% would increase annual revenue per sheep by around USD 2.5. At zero variable production costs, this figure would represent the upper limit for any investment in YSM reduction to be financially worthwhile while at 15% production cost this figure would reduce to USD 2.13. From a collective point of view, extrapolation of the USD 2.5 YSM revenue loss per sheep to 24.7 million sheep in the MCL system in Ethiopia (15) yields an expected increase in annual revenue of USD 61.8 million from reducing YSM from 20% to 10%. According to Jemberu et al. (15), 75% of MCL sheep flocks consist of five or less animals. Thus, for most sheep keepers, any investment in YSM reduction that exceeds USD 12.5 per year, or USD 1.04 per month, would not be financially worthwhile, unless it entails collateral benefits, which have not been considered in this analysis. As improved feeding of ewes before parturition is part of the intervention packages, improved fertility might be one potential collateral benefit worth assessing. On the other hand, fixed costs, such as (improving) housing, might quickly make the intervention package unattractive for farmers with small flocks. Even if financially worthwhile, other considerations are likely to also influence adoption of “better” practices by smallholder farmers relying on multiple income sources (21). More detailed socio-economic research covering more than the sheep enterprise would be required to accurately assess smallholders' “willingness to invest” in reducing YSM.

The analysis examines the cost of YSM to farmers only and omits its cost to value chain actors and consumers. On the one hand, increased YSM results in foregone value added along the value chain, while on the other, increased supply results in lower consumer prices, transferring some of the benefits of reduced YSM from producers to consumers (22). Again, extrapolating from Jemberu et al.'s (15) figure of 24.7 million sheep in MCL systems in Ethiopia, and assuming farmers would maintain the national average annual flock growth of slightly above 10%, around 740,000 additional sheep would be marketed per year if YSM were reduced from 20% to 10% (estimated from Supplementary Table 1). This represents a 13% increase of sheep in the market which is likely to have non-negligible consequences for value-chain actors and consumers. Both the direct effects on producer households and the secondary downstream effects need to be considered and quantified when planning public sector engagement in improving livestock productivity at farm level.

In conclusion, this case study provides three generalizable insights: (i) the “cost of YSM” is contingent on other production parameters, (ii) the law of diminishing returns also applies to YSM reduction, and (iii) the “visible” losses in form of dead offspring only represent a fraction of the total systemic cost, which is complemented by the “invisible” loss from revenue/profit forgone had the animal survived. Caution should, however, be exercised with respect to the specific monetary results of the analysis. We have reservations about some of the production parameter values, e.g., the large difference in mortality between sub(-adult) females and their male counterparts or the parturition rate of 60%. A review of productive and reproductive performances of indigenous sheep in Ethiopia by Ayele and Urge (1) reports lambing intervals to be in the order of seven to 10 months, which suggest a parturition rate far above 60%. Also, the stark difference in prices between adult males and females (more than double at the same live weight) appears questionable and the high price of male adults substantially inflates the estimated cost of YSM. Thus, for more accurate and site-specific assessments of the cost of YSM it is essential to replicate the analysis with local production parameter values and prices.

## Data availability statement

The original contributions presented in the study are included in the article/Supplementary material, further inquiries can be directed to the corresponding author.

## Author contributions

JO: Formal analysis, Writing – original draft, Writing – review & editing. CS: Writing – original draft, Writing – review & editing. FA: Writing – original draft, Writing – review & editing. GS: Writing – original draft, Writing – review & editing. JW: Writing – original draft, Writing – review & editing. BM: Funding acquisition, Supervision, Validation, Writing – original draft, Writing – review & editing.
