# Supplementary material for: Estimating the cost of young stock mortality in livestock systems—An application to sheep farming in Ethiopia
Source: Front Vet Sci. 2024 Jul 24;11:1389303. doi: 10.3389/fvets.2024.1389303 (PMC11303338; doi:10.3389/fvets.2024.1389303)
Supplement: Supplementary file 1 [file Data_Sheet_1.PDF]

Supplementary Material

Table S1. Production parameter values and market prices used to estimate the annual offtake value from sheep in mixed crop-livestock (MCL) systems in Ethiopia, taken from Jemberu et al. (2022)

Production Parameter	Value	Output	Price	
			Birr	USD
Parturition rate (%)	59.0	Lamb, female	1,200	28
Prolificacy (no)	1.27	Lamb, male	1,250	29
Lamb mortality (%)	20.0[1]	Sub-adult, female	1,925	45
Female sub-adult & adult mortality (%)	4.0	Sub-adult, male	2,600	61
Male sub-adult & adult mortality (%)	12.0	Adult, female	2,825	66
Offtake sub-adult females (%)	4.0	Adult, male	6,000	148
Offtake sub-adult males (%)	54.0	Manure (t)	500	12
Liveweight of lambs (kg)	12.0			
Liveweight of sub-adults (kg)	16.0			
Liveweight of adults (kg)	28.0			

[1] 18% in Jemberu et al. (2022)

Table S2. Simulated demographics and offtake of a sheep flock of 100 animals at annual 10.2% growth using production parameter values and prices from Jemberu et al. (2022) at 20% ('baseline'), 10% and 0% lamb mortality risk.

	**20% Lamb mortality**		**10% Lamb mortality**		**0% Lamb mortality**	
	Nr	%	Nr	%	Nr	%
**Inventory**						
Adult / breeding females	60.1	60.1	58.2	58.2	56.4	56.4
Adult / breeding males	6.1	6.1	6.6	6.6	7.1	7.1
Other females	18.0	18.0	18.8	18.8	19.6	19.6
Other males	15.8	15.8	16.4	16.4	17.0	17.0
**Total**	**100.0**		**100.0**		**100.0**	
**Deaths**						
Adults / breeders	3.3	22.8	3.3	33.0	3.3	57.1
Sub-adults	2.1	14.5	2.3	22.9	2.5	42.9
Lambs	9.1	62.7	4.4	44.0	0.0	0.0
**Total**	**14.6**		**10.0**		**5.8**	
**Live animal offtake**						
Adult / breeding females	7.3	32.1	9.0	34.8	10.5	36.8
Adult / breeding males	5.3	23.1	5.7	22.2	6.1	21.5
Sub-adult females	0.7	3.1	0.8	2.9	0.8	2.8
Sub-adult males	9.5	41.7	10.3	40.1	11.1	38.8
Lambs	0.0	0.0	0.0	0.0	0.0	0.0
**Total**	**22.7**		**25.7**		**28.6**	
**Value of flock growth and offtake (USD)**						
Flock growth	617.4	24.3	621.0	22.4	622.5	20.9
Live animal sales	1,818.5	71.4	2,042.2	73.7	2,253.8	75.5
Manure	109.8	4.3	109.1	3.9	108.4	3.6
**Total**	**2,545.7**		**2,772.3**		**2,984.7**	
Per head	***24.2***		***26.4***		***28.4***	
Per breeding female	***40.3***		***45.3***		***50.4***	
Per 100kg feed DM	***11.0***		***12.1***		***13.1***	

Table S3. Absolute (USD, blue area) and relative (%, grey area) change in revenue per animal through changes in lamb mortality risk at 59% parturition rate**.**

Lamb Mortality (%)	30.0	25.0	20.0	15.0	10.0	5.0	0.0
**30.0**		1.35	2.66	3.94	5.18	6.40	7.56
**25.0**	6.18		1.31	2.59	3.84	5.05	6.21
**20.0**	12.19	5.66		1.28	2.52	3.74	4.90
**15.0**	18.05	11.18	5.22		1.25	2.46	3.62
**10.0**	23.76	16.56	10.31	4.84		1.22	2.38
**5.0**	29.33	21.81	15.28	9.56	4.50		1.16
**0.0**	34.66	26.82	20.03	14.07	8.81	4.12	

Table S4. Simulated demographics and offtake of a sheep flock of 100 breeding females at 100% parturition rate using all other production parameter values and prices from Jemberu et al. (2022) at 20% ('baseline'), 10% and 0% lamb mortality risk.

	**20% Lamb mortality**		**10% Lamb mortality**		**0% Lamb mortality**	
	Nr	%	Nr	%	Nr	%
**Inventory**						
Adult / breeding females	100.0	47.5	100.0	45.7	100.0	44.1
Adult / breeding males	10.5	5.0	10.5	4.8	10.5	4.6
Other females	53.5	25.4	58.0	26.5	62.4	27.5
Other males	46.6	22.1	50.2	23.0	53.7	23.7
**Total**	**210.6**		**218.7**		**226.6**	
**Deaths**						
Adults / breeders	5.4	16.7	5.4	23.7	5.4	41.3
Sub-adults	6.1	19.0	6.9	30.3	7.7	58.7
Lambs	20.8	64.2	10.5	46.0	0.0	0.0
**Total**	**32.3**		**22.8**		**13.1**	
**Live animal offtake**						
Adult / breeding females	42.7	47.4	48.5	47.6	54.4	47.8
Adult / breeding males	17.9	19.9	20.3	19.9	22.7	19.9
Sub-adult females	2.0	2.3	2.3	2.2	2.5	2.2
Sub-adult males	27.4	30.5	30.9	30.3	34.3	30.1
Lambs	0.0	0.0	0.0	0.0	0.0	0.0
**Total**	**90.0**		**102.0**		**113.9**	
**Offtake value (USD)**						
Live animals	7,050.0	97.2	7,988.0	97.5	8,926.0	97.7
Manure	201.1	2.8	206.7	2.5	212.4	2.3
**Total**	**7,251.1**		**8,194.7**		**9,138.3**	
Per head	***34.4***		***37.5***		***40.3***	
Per breeding female	***72.5***		***81.9***		***91.4***	
Per 100kg feed DM	***17.1***		***18.9***		***20.5***	

Table S5. Absolute (USD, blue area) and relative (%, grey area) change in revenue per animal through changes in lamb mortality risk**.**

Lamb Mortality (%)	30.0	25.0	20.0	15.0	10.0	5.0	0.0
**30.0**		1.66	3.26	4.81	6.30	7.75	9.12
**25.0**	5.33		1.60	3.15	4.64	6.09	7.46
**20.0**	10.47	4.88		1.55	3.04	4.49	5.86
**15.0**	15.43	9.59	4.49		1.49	2.94	4.31
**10.0**	20.23	14.15	8.83	4.16		1.45	2.82
**5.0**	24.87	18.55	13.03	8.17	3.86		1.37
**0.0**	29.27	22.73	17.02	11.99	7.52	3.53	
